# Supplementary figures and images for: Seasonal variations in gut microbiota and disease course in patients with inflammatory bowel disease
Source: PLoS One. 2023 Apr 18;18(4):e0283880. doi: 10.1371/journal.pone.0283880 (PMC10112787; doi:10.1371/journal.pone.0283880)

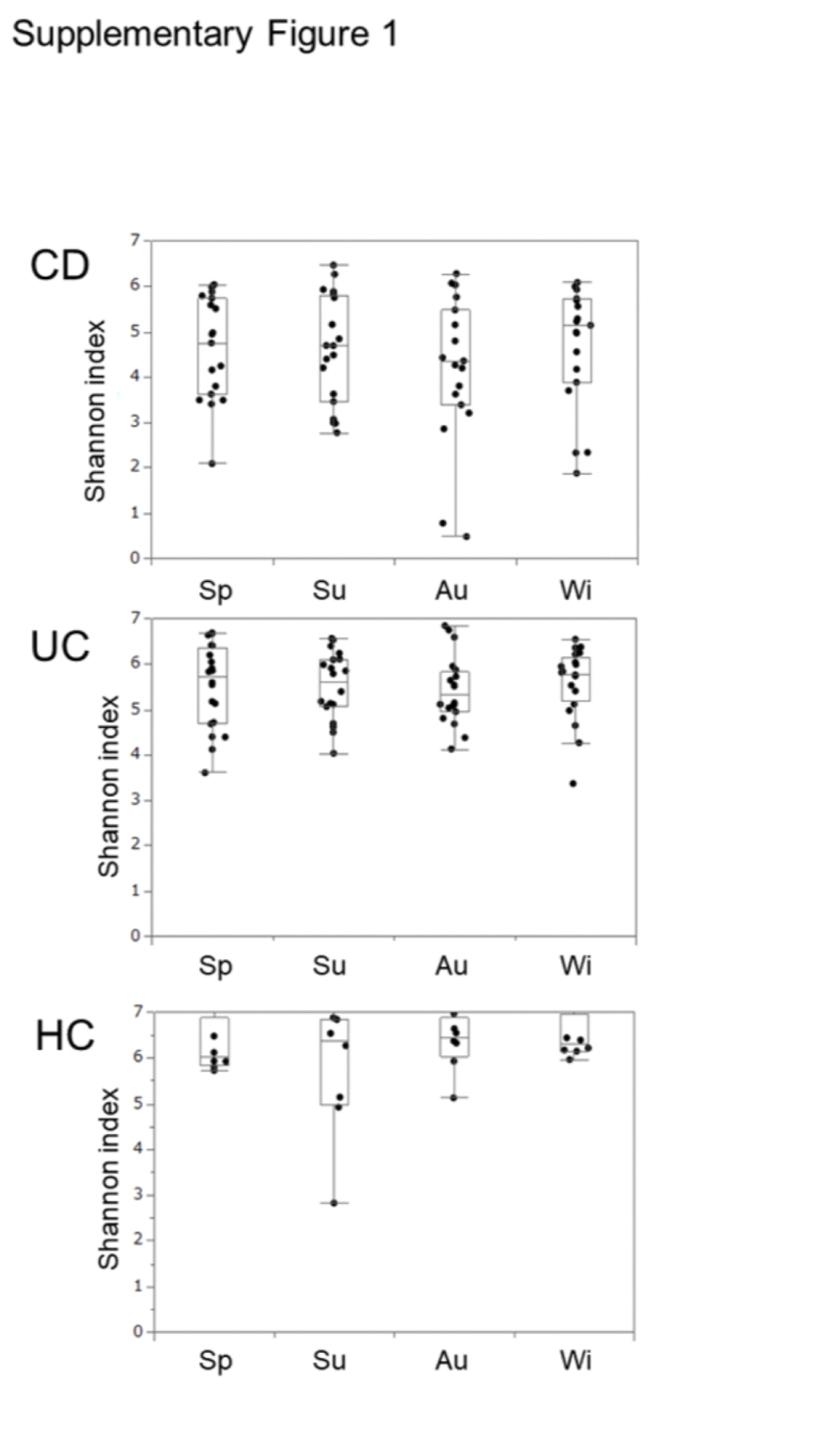

Supplement: S1 Fig — The vertical axis shows the Shannon index for each season, and the horizontal axis shows the season. Bonferroni correction was performed with a paired test for correspondence. No significant difference was found. (TIF) [file pone.0283880.s001.tif]

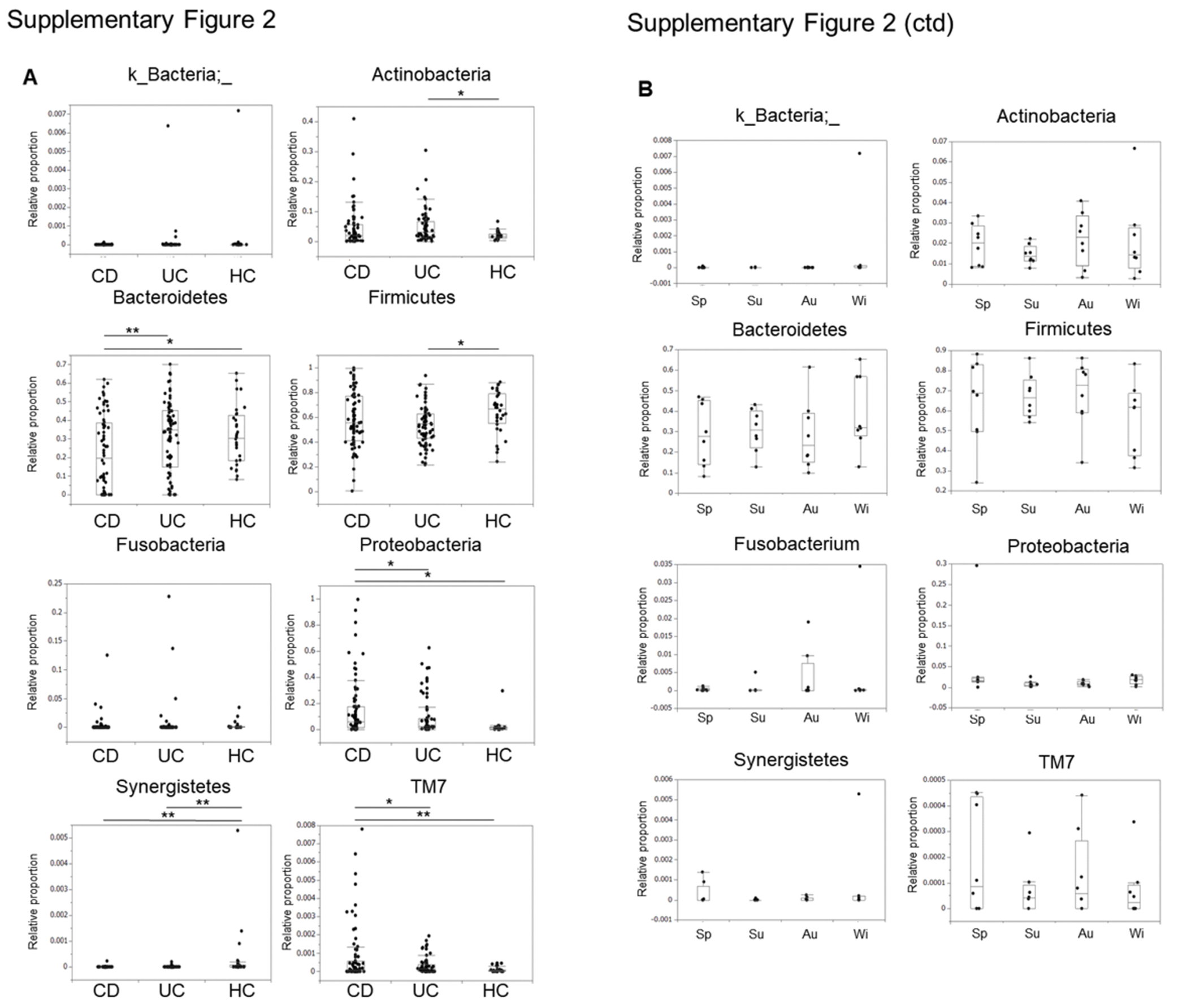

Supplement: S2 Fig — (A) Comparison of relative abundances at the phylum level in the four seasons between the IBD patients and HCs. The vertical axis shows the relative abundance, and the horizontal axis indicates each disease. The p value indicates statistical significance according to Tukey’s HSD test *: p < 0.05, **: p < 0.01 (B) Seasonal changes at the phylum level in the HCs. The eight main phyla with the largest proportions were examined. Phyla are listed in alphabetical order. There was no significant seasonal variation in any of the phyla. Bonferroni correction was performed with a paired test for correspondence, and a p value less than 0.0083 was considered significant. (TIF) [file pone.0283880.s002.tif]

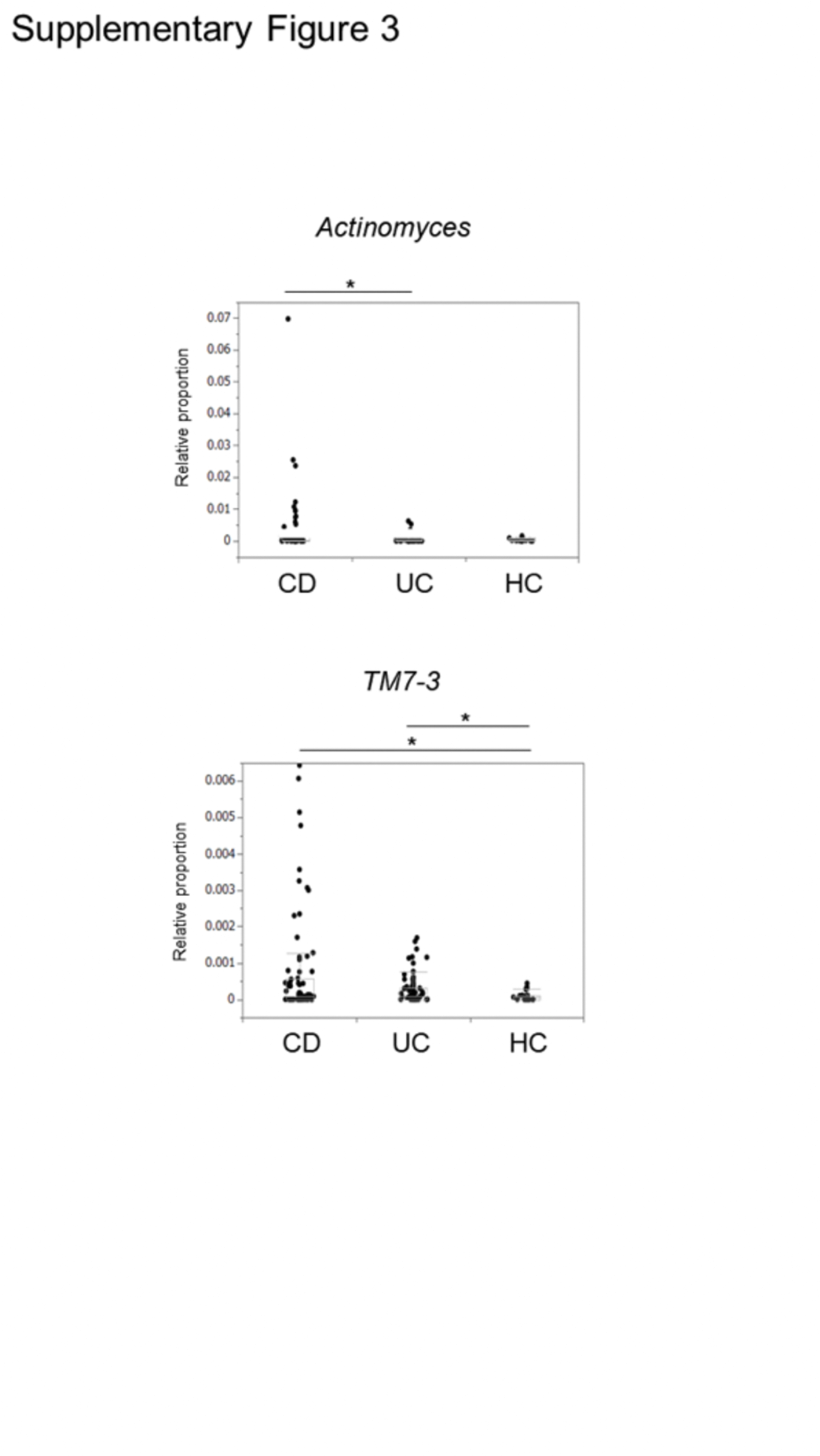

Supplement: S3 Fig — The vertical axis shows the relative abundance, and the horizontal axis indicates each disease group. Bacteria belonging to the phyla Actinobacteria and TM7 (genus level) showed seasonal variations in IBD patients. The p value indicates statistical significance according to Tukey’s HSD test. * indicates p < 0.05. (TIF) [file pone.0283880.s003.tif]

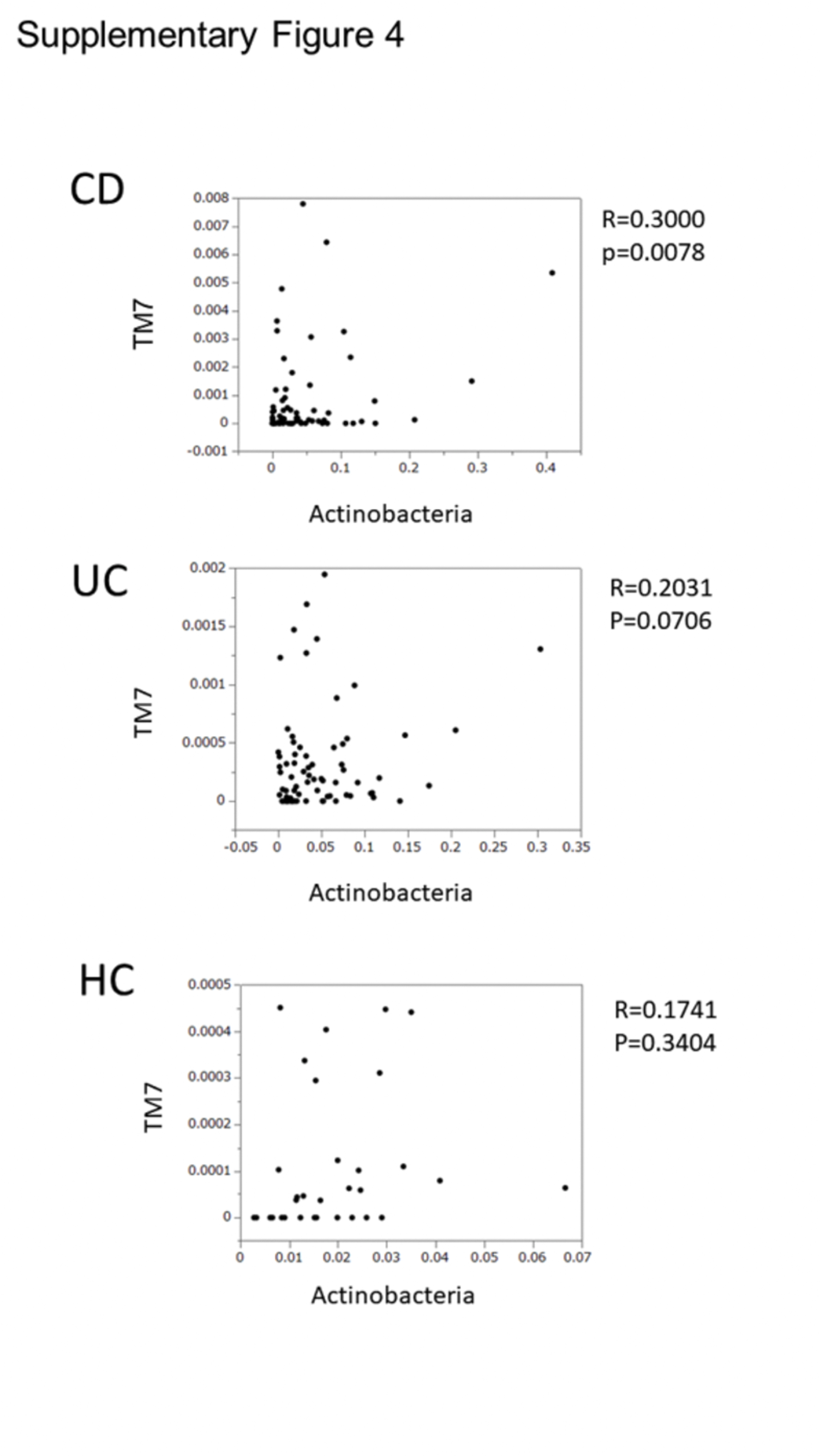

Supplement: S4 Fig — Both the vertical and horizontal axes show the relative abundance. (TIF) [file pone.0283880.s004.tif]

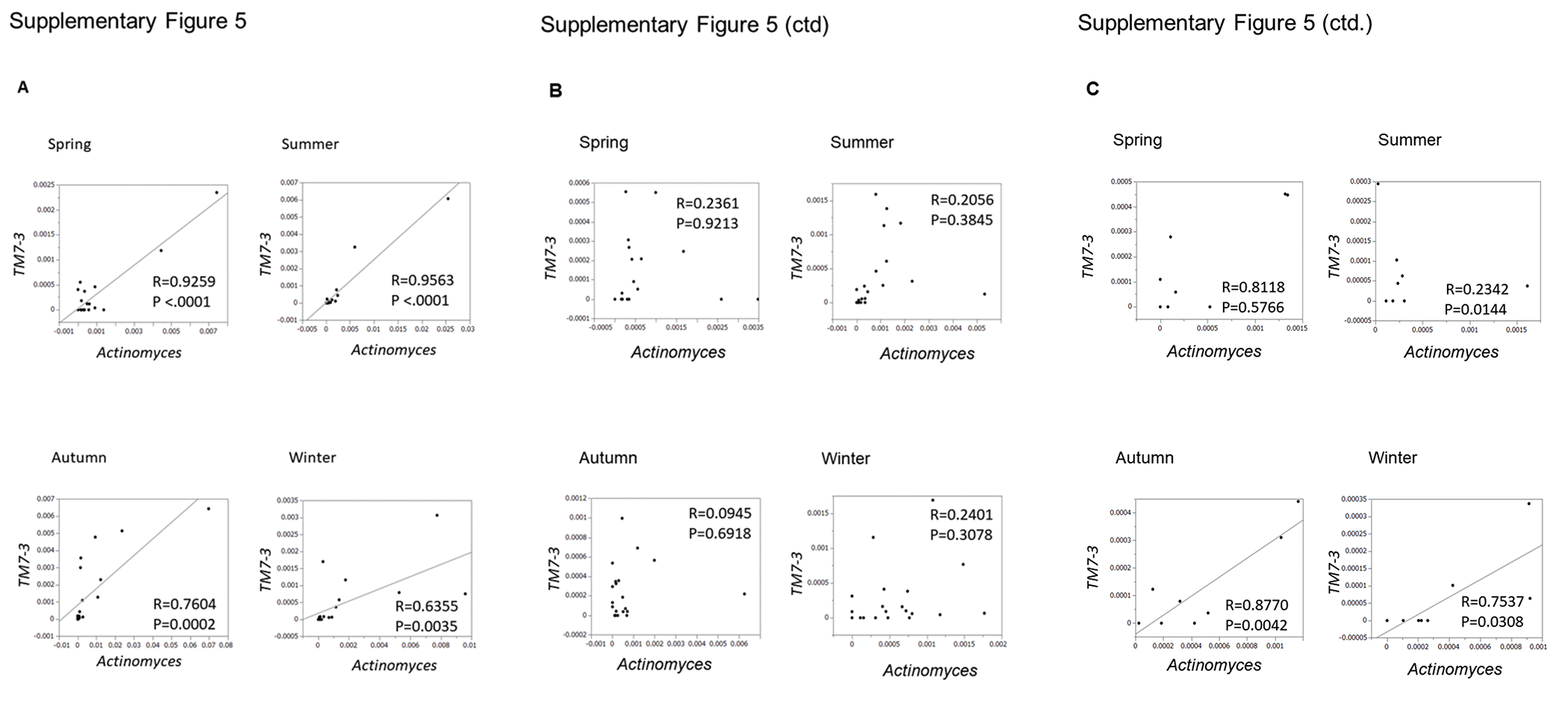

Supplement: S5 Fig — Correlations of these bacteria in the CD patients (A), UC patients (B) and HCs (C) are shown. (TIF) [file pone.0283880.s005.tif]

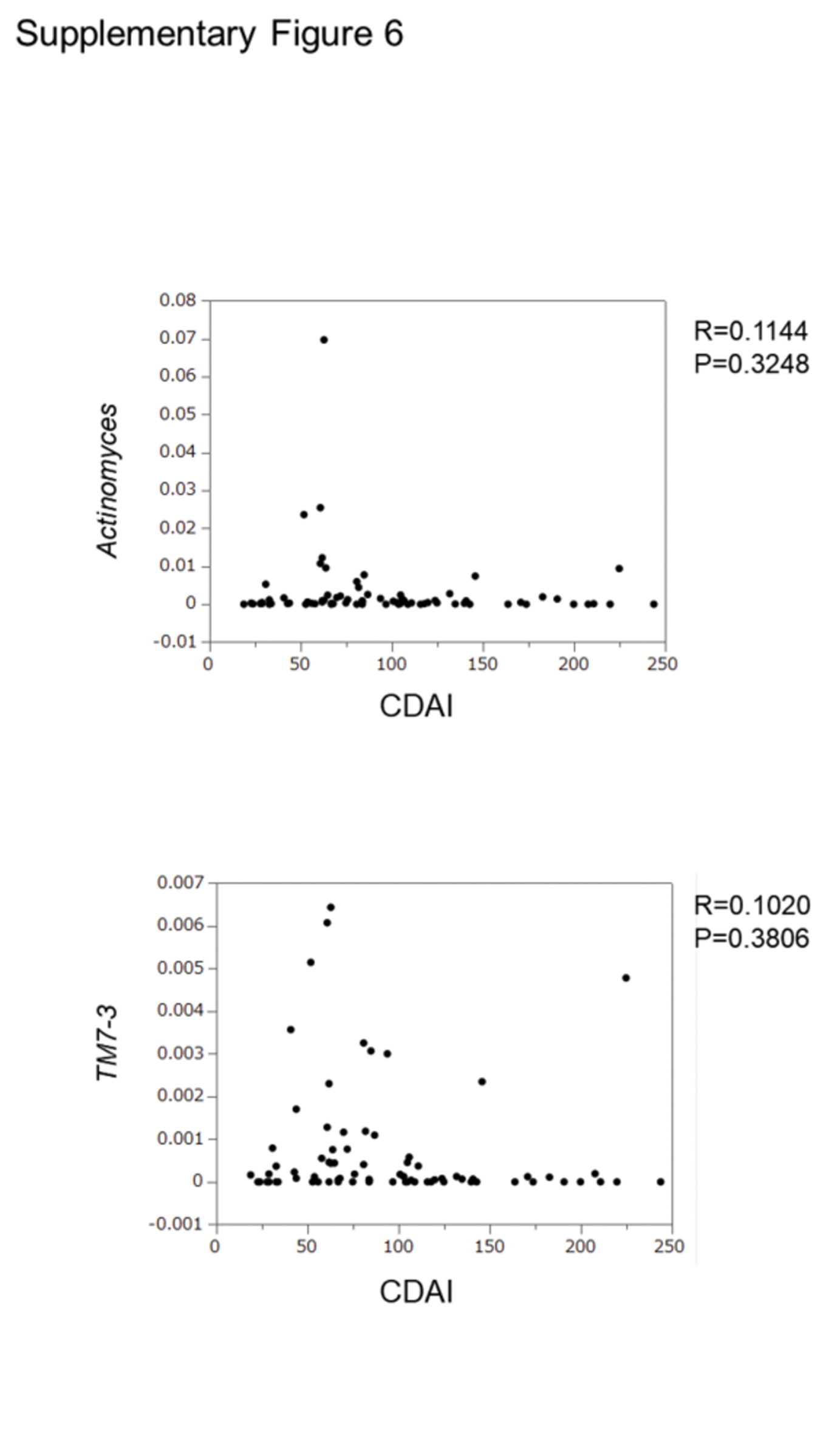

Supplement: S6 Fig — There was no correlation between Actinomyces or TM7-3 and CDAI. (TIF) [file pone.0283880.s006.tif]

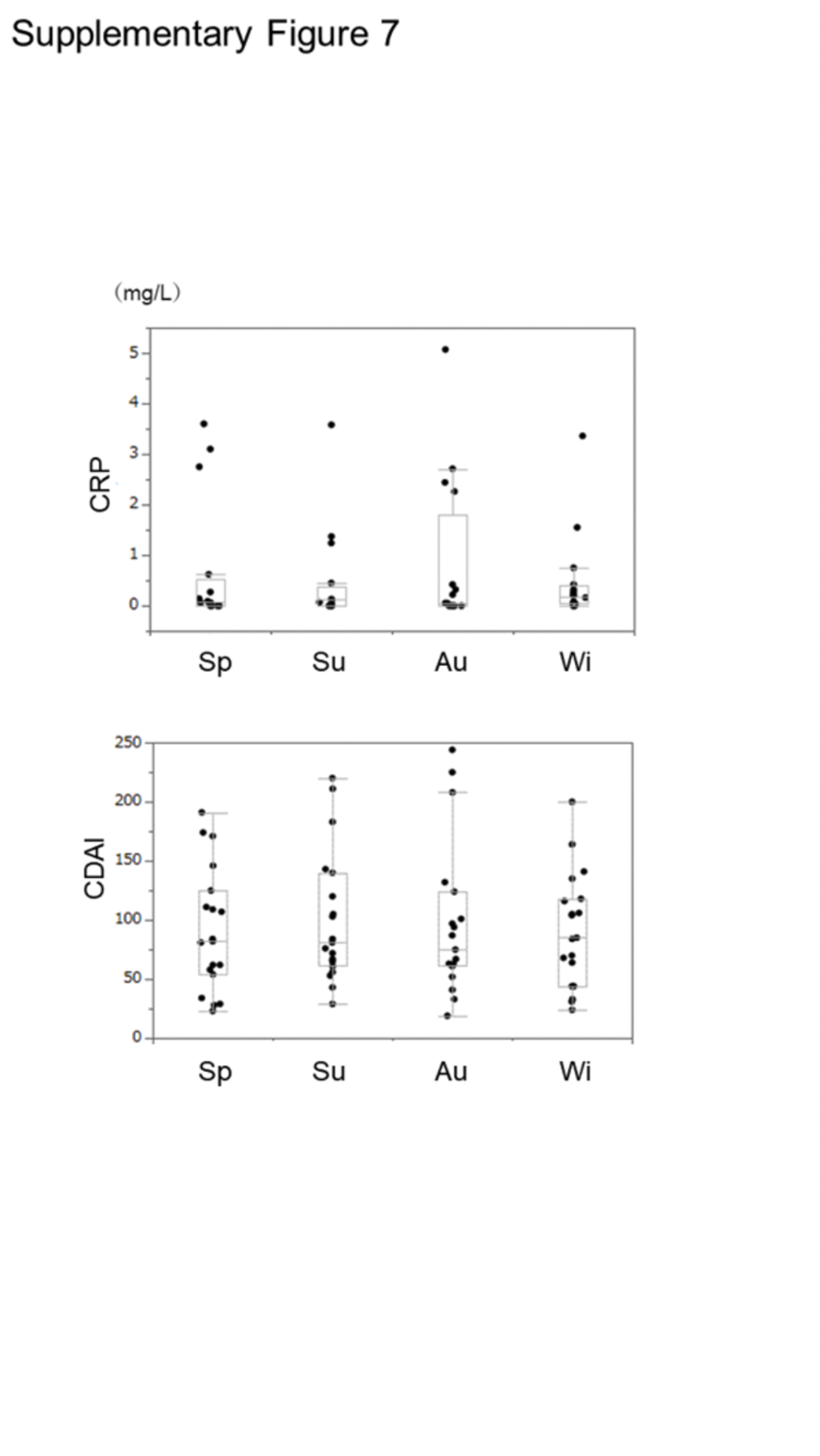

Supplement: S7 Fig — The vertical axis shows C-reactive protein (CRP) (A) and CDAI (B), and the horizontal axis shows the season (*Sp: Spring, Su: Summer, Au: Autumn, Wi: Winter). Bonferroni correction was performed with a paired test for correspondence, and a p value of less than 0.0083 was considered significant. (TIF) [file pone.0283880.s007.tif]
