# Supplementary material for: Seasonal variations in gut microbiota and disease course in patients with inflammatory bowel disease
Source: PLoS One. 2023 Apr 18;18(4):e0283880. doi: 10.1371/journal.pone.0283880 (PMC10112787; doi:10.1371/journal.pone.0283880)
Supplement: Attachment — Submitted filename: recomment.docx [file pone.0283880.s008.docx]

This manuscript examined the fecal microbiota of patients with inflammatory bowel disease, revealing seasonal changes in the gut microbiota. IBD is an urgent health issue, so this manuscript is timely. Overall, the manuscript is well written and the conclusions are fully supported by the findings. However, more polish should be considered. In my opinion, suitable revision should be conducted by the authors to ensure this manuscript can meet the standard quality of the PLoS One.

1. This paper mainly studies the influence of seasonal changes on intestinal flora, temperature and the diet may be the important factors, etc. Although all experimental materials were obtained in the same hospital, but they cannot represent the comparability of patients' living environment and eating habits, so it is necessary to describe the relevant background in detail.
2. The comment HV represented by the blue circle in Figure1 B should be changed to HC.
3. The format of references is not uniform, for example, in the line 556“de Vries SAG”. So all the references should be carefully verified.
4. The pictures in the full text are not clear.
5. Latin words such as Actinobacteria, etc. should be italicize.
6. The abundance of phyla Actinobacteria and TM7 in autumn was compared with spring and winter, why the summer was not mentioned?
7. Line 161-162“The median (range) ages of CD and UC 162 patients and HCs were 44 (32-55), 47 (44-57), and 33 (32-43) years, respectively.”

The age of the test group and the control group are also quite different. Do you consider the influence of age on the regulating ability of the flora, that is, the younger people have better regulating ability, so they do not get sick? Why not choose a control group of the similar age?

8. Line 284, The annotation format of references is incorrect.
9. In the full text and in the chart, p values are not written in standard at many places: line 186, p<.005 is changed to p<0.005; Change p<.0001 to p<0.0001 in line 187; Change p<.0001 to p<0.0001 in line 191; Change p<.0083 to p<0.0083 in line 220; Change p<.0083 to p<0.0083 at 228; In Fig5, change p<.0001 to p<0.0001 on the right side of figure CD; In S5A, p<.0001 is changed to p<0.0001
10. Supplementary chart missing: The chart mentioned in 250 lines of S5C Fig is missing.
